# Mapping the Laminin Receptor Binding Domains of *Neisseria meningitidis* PorA and *Haemophilus influenzae* OmpP2

**DOI:** 10.1371/journal.pone.0046233

**Published:** 2012-09-25

**Authors:** Noha M. Abouseada, Mahde Saleh A. Assafi, Jafar Mahdavi, Neil J. Oldfield, Lee M. Wheldon, Karl G. Wooldridge, Dlawer A. A. Ala'Aldeen

**Affiliations:** Molecular Bacteriology and Immunology Group, Centre for Biomolecular Sciences, University of Nottingham, Nottingham, United Kingdom; Health Protection Agency, United Kingdom

## Abstract

*Neisseria meningitidis*, *Haemophilus influenzae* and *Streptococcus pneumoniae* are major bacterial agents of meningitis. They each bind the 37/67-kDa laminin receptor (LamR) via the surface protein adhesins: meningococcal PilQ and PorA, *H. influenzae* OmpP2 and pneumococcal CbpA. We have previously reported that a surface-exposed loop of the R2 domain of CbpA mediates LamR-binding. Here we have identified the LamR-binding regions of PorA and OmpP2. Using truncated recombinant proteins we show that binding is dependent on amino acids 171–240 and 91–99 of PorA and OmpP2, respectively, which are predicted to localize to the fourth and second surface-exposed loops, respectively, of these proteins. Synthetic peptides corresponding to the loops bound LamR and could block LamR-binding to bacterial ligands in a dose dependant manner. Meningococci expressing PorA lacking the apex of loop 4 and *H. influenzae* expressing OmpP2 lacking the apex of loop 2 showed significantly reduced LamR binding. Since both loops are hyper-variable, our data may suggest a molecular basis for the range of LamR-binding capabilities previously reported among different meningococcal and *H. influenzae* strains.

## Introduction

Invasive disease caused by *Neisseria meningitidis* (meningococcus), *Haemophilus influenzae* and *Streptococcus pneumoniae* (pneumococcus) remains an important cause of morbidity and mortality worldwide, despite the use of antibiotics and the introduction of effective conjugate polysaccharide vaccines that target some disease-associated serogroups [1]. However, there is no currently licensed vaccine effective against serogroup B meningococcus, the dominant serogroup in much of the developed world [2]. Similarly, there are no vaccines against non-type B *H. influenzae* and the vaccines available against pneumococcus only provide protection against a limited subset of the plethora of recognized serogroups [1].

The human 37/67-kDa laminin receptor (LamR) is a highly conserved, multi-functional protein [3]. The relationship between the 37-kDa and 67-kDa forms is not completely understood, but the former is thought to mature into one or more homo or heterodimeric 67-kDa forms [4], [5], [6]. LamR was initially identified as a cell surface receptor for the extracellular matrix molecule laminin [7], [8], [9]. LamR is important for cell adhesion to the basement membrane and is also implicated in tumour cell metastasis [10]. LamR has additional roles in intracellular signaling [11], ribosomal activity [12] and cell viability [13]. LamR can also migrate to the nucleus where it can bind the histones H2A, H2B and H4, although the significance of this is not known [14].

Various neurotropic bacteria and viruses use LamR as a receptor to bind host microvascular endothelial cells. Viruses including Sindbis [15], dengue [16], adeno-associated [17], tick-borne encephalitis [18] and Venezuelan equine encephalitis viruses [19] bind LamR. The cytotoxic necrotizing factor toxin (CNF1) of *Escherichia coli* [20] and the cellular prion protein PrP [21] also interact with LamR.

Recently we showed that binding to LamR is a common mechanism employed by *S. pneumoniae*, *N. meningitidis* and *H. influenzae* to adhere to human brain microvascular endothelial cells [22]. The bacterial ligands responsible for LamR binding were identified as pneumococcal CbpA, meningococcal PilQ and PorA and *H. influenzae* OmpP2 [22]. All are abundant, multi-functional proteins with surface-exposed loop structures. In particular, meningococcal PorA and *H. influenzae* OmpP2 share many characteristics, despite showing limited sequence similarity. Both are homo-trimeric outer membrane porins with amphipathic β-barrel structures forming sixteen membrane-spanning β-strands separating more variable sequences forming eight surface exposed loops [23], [24]. In the case of OmpP2, most sequence variability occurs in the second, fourth, fifth and eighth loops (known as variable regions 1, 2, 3 and 4, respectively) [25], [26], whereas in PorA most variation occurs in the first, fourth and fifth loops (variable regions 1, 2 and 3, respectively) [27], [28]. Indeed sequence variability of PorA is the basis of the meningococcal subtyping strategy [29], [30], [31].

As immunodominant antigens and targets for serum bactericidal antibodies, PorA and OmpP2 have been studied as potential vaccine targets [32], [33]. However, bactericidal antibodies elicited by such vaccines predominately target the variable extra-cellular loops [23], [27], [34]. Therefore, generating an immune response against one antigen does not generally confer protection against strains with heterologous antigens.

Previously, we showed that the highly conserved surface-exposed loop (residues ^391^EPRNEEK^397^) linking the second and third anti-parallel α-helices of the R2 domain of pneumococcal CbpA mediated LamR-binding [22]. Here, using recombinant derivatives of PorA and OmpP2, synthetic peptides corresponding to their extracellular loops, and bacterial strains expressing LamR ligands in which specific extracellular loops were deleted, we identify the fourth and second extra-cellular loops of PorA and OmpP2, respectively, as the LamR-binding domains of these proteins.

## Results

### Amino acids 171–240 of recombinant PorA exhibit LamR-binding activity

**Figure 1:**
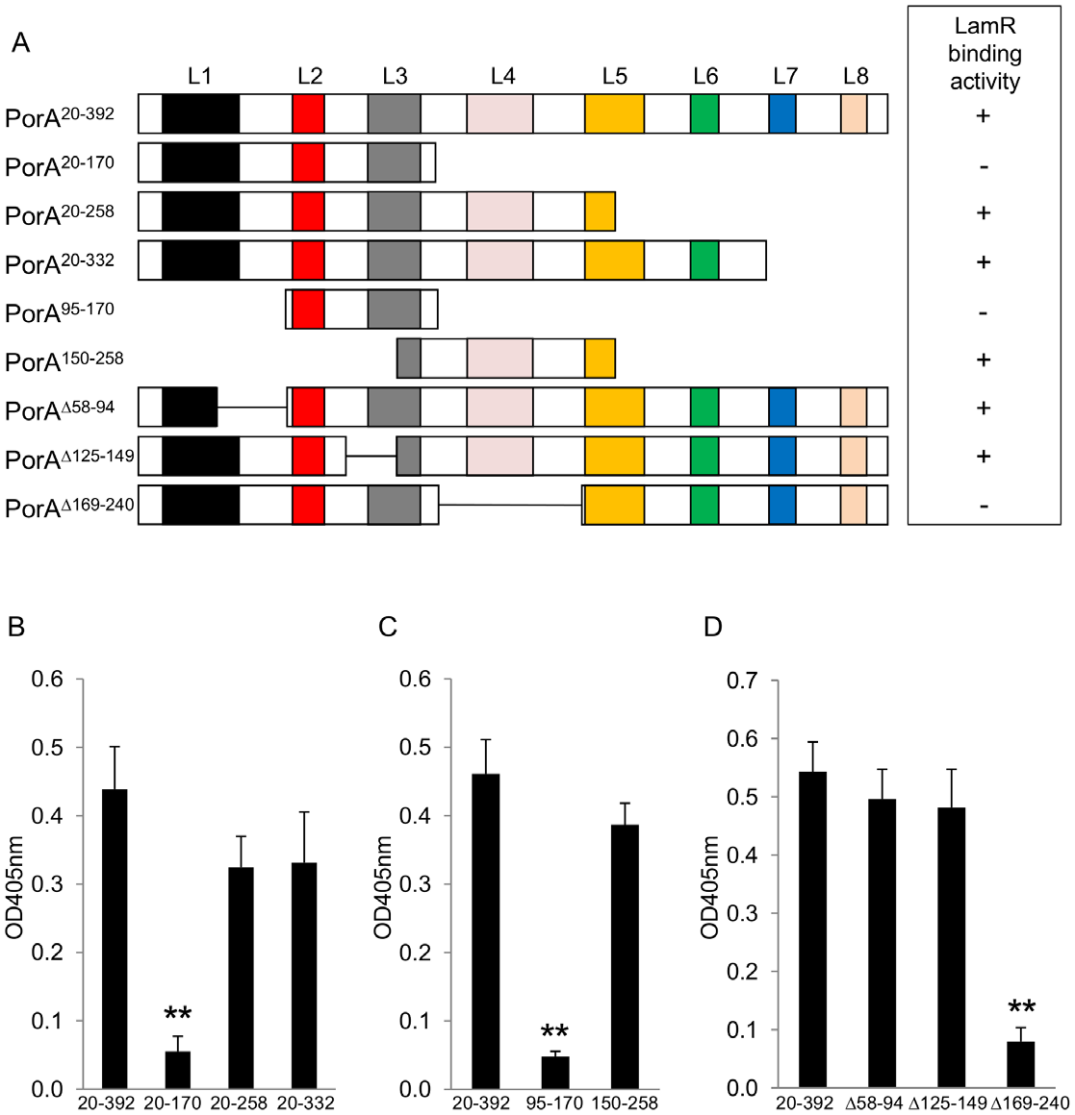
Amino acids 171–240 of recombinant PorA exhibit LamR-binding activity. (A) Schematic showing the recombinant derivatives of *N. meningitidis* MC58 PorA utilized in this study and a summary of their respective LamR-binding activities. L1–L8 denotes the eight extra-cellular loops based on the model of van der Ley *et al.* [23]. (B) Binding of LamR to the solid phase antigens PorA^20–392^, PorA^20–170^, PorA^20–258^ and PorA^20–332^. (C) Binding of LamR to PorA^20–392^, PorA^95–170^ and PorA^150–258^. (D) Binding of LamR to PorA^20–392^, PorA^Δ58–94^, PorA^Δ125–149^ and PorA^Δ169–240^. Data shown in each panel are means from three independent experiments; in each experiment each sample was tested in triplicate. ** *p*<0.01 compared to binding with matching PorA^20–392^ samples. Error bars indicate SEM.

To identify the regions of *N. meningitidis* MC58 PorA that elicit LamR binding activity, recombinant PorA (minus the 19 amino acid cleavable N-terminal signal peptide; PorA^20–392^) and three sub-fragments of PorA (PorA^20–170^, PorA^20–258^ and PorA^20–332^; Fig. 1A) were expressed and purified. LamR-binding activity of PorA^20–170^ was significantly reduced compared to PorA^20–392^ in ELISA assays (Fig. 1B). This suggested that the LamR-binding region was localized between amino acids 171–258. The sub-fragments PorA^95–170^ and PorA^150–258^ (Fig. 1A) were subsequently tested in ELISA assays; binding of PorA^150–258^ was not significantly different from PorA^20–392^ (Fig. 1C). As expected, PorA^95–170^ showed significantly reduced LamR-binding when compared to PorA^20–392^.

Next, derivatives of PorA^20–392^ were expressed in which the residues spanning loop 4 were removed (PorA^Δ169–240^). This derivative showed significantly less LamR-binding activity than PorA^20–392^ (Fig. 1D). In contrast, control PorA derivatives lacking parts of loops 1 (PorA^Δ58–94^) or 3 (PorA^Δ125–149^) exhibited similar LamR binding to PorA^20–392^, providing additional confirmation that these regions are not required for optimal LamR binding (Fig. 1D). Taken together these results indicated that the LamR-binding domain of MC58 PorA lies within amino acids 171–240 and was likely localized to the fourth extra-cellular loop which comprises amino acids 185–218.

### Amino acids 91–99 of recombinant OmpP2 exhibit LamR-binding activity

**Figure 2:**
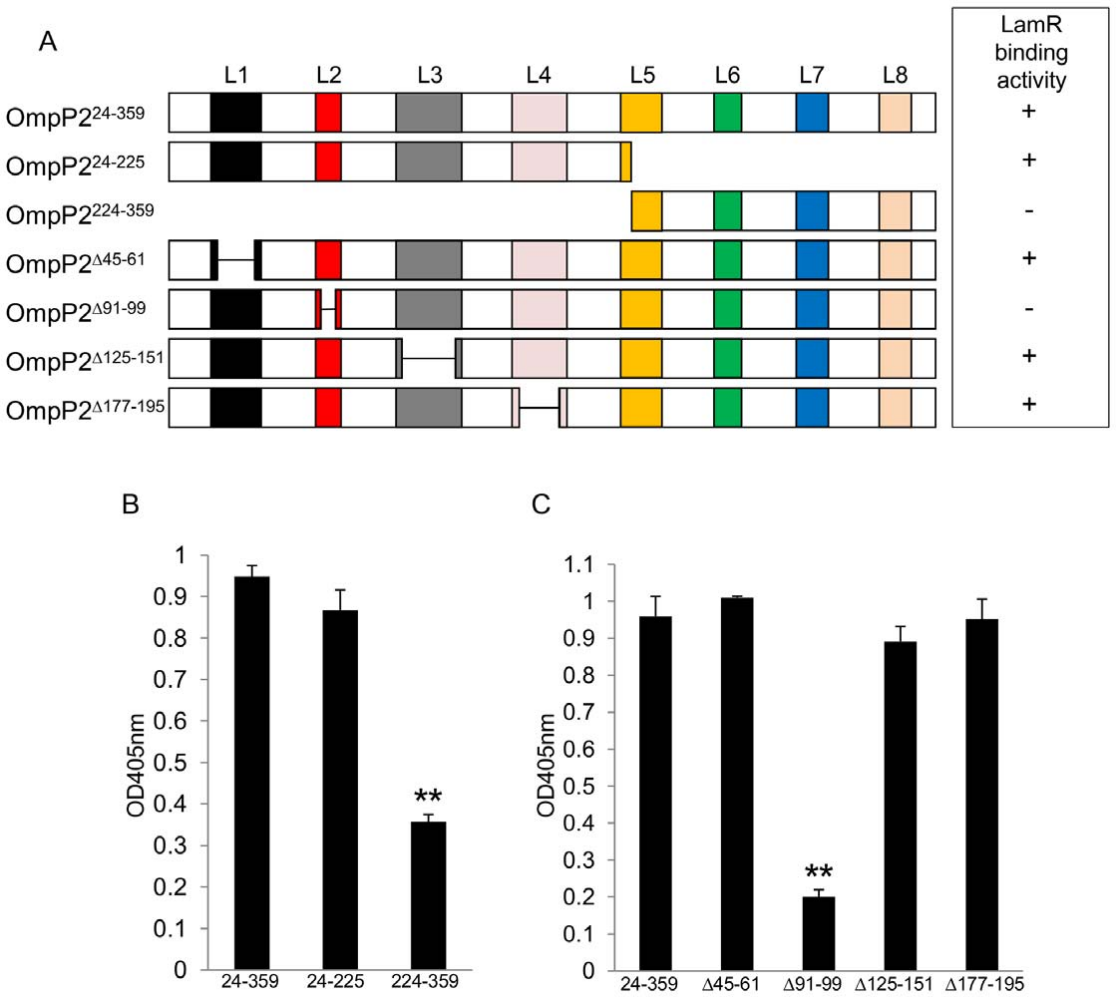
Amino acids 91–99 of recombinant OmpP2 exhibit LamR-binding activity. (A) Schematic showing the recombinant derivatives of *H. influenzae* Rd KW20 OmpP2 utilized in this study and a summary of their respective LamR-binding activities. L1–L8 denotes the eight extra-cellular loops based on the model of Srikumar *et al.* [24]. (B) Binding of LamR to the solid phase antigens OmpP2^24–359^, OmpP2^24–225^ OmpP2^224–359^. (C) Binding of LamR to OmpP2^24–359^, OmpP2^Δ45–61^, OmpP2^Δ91–99^, OmpP2^Δ125–151^ and OmpP2^Δ177–195^. Data shown in each panel are means from three independent experiments; in each experiment each sample was tested in triplicate. ** *p*<0.01 compared to binding with matching OmpP2^24–359^ samples. Error bars indicate SEM.

In a similar approach to that used to identify the LamR-binding region of *N. meningitidis* MC58 PorA, recombinant *H. influenzae* Rd KW20 OmpP2 (minus the first 23 N-terminal amino acids; OmpP2^24–359^) and two sub-fragments (OmpP2^24–225^ and OmpP2^224–359^; Fig. 2A) were expressed and purified. Of the two sub-fragments, OmpP2^224–359^ showed significantly less LamR-binding compared to OmpP2^24–359^ (Fig. 2B). This suggested that the LamR-binding region was localized between amino acids 24–224.

We hypothesized that the LamR-binding activity was localized to one of the extra-cellular loops of OmpP2 between amino acids 24–224; four derivatives of OmpP2^24–359^ were expressed and purified in which the residues spanning the apical regions of loops 1, 2, 3 or 4 were removed. The derivative lacking loop 2 (OmpP2^Δ91–99^) showed significantly less LamR binding activity compared to OmpP2^24–359^ (Fig. 2C), whilst the other derivatives lacking loops 1, 3 or 4 showed no significant reductions in binding. These results indicated that the LamR-binding domain of OmpP2 lies within amino acids 91–99, corresponding to the apical region of the second extra-cellular loop, which comprises amino acids 88–102.

### Synthetic peptides corresponding to PorA loop 4 and OmpP2 loop 2 bind LamR and block ligand-LamR binding

**Figure 3:**
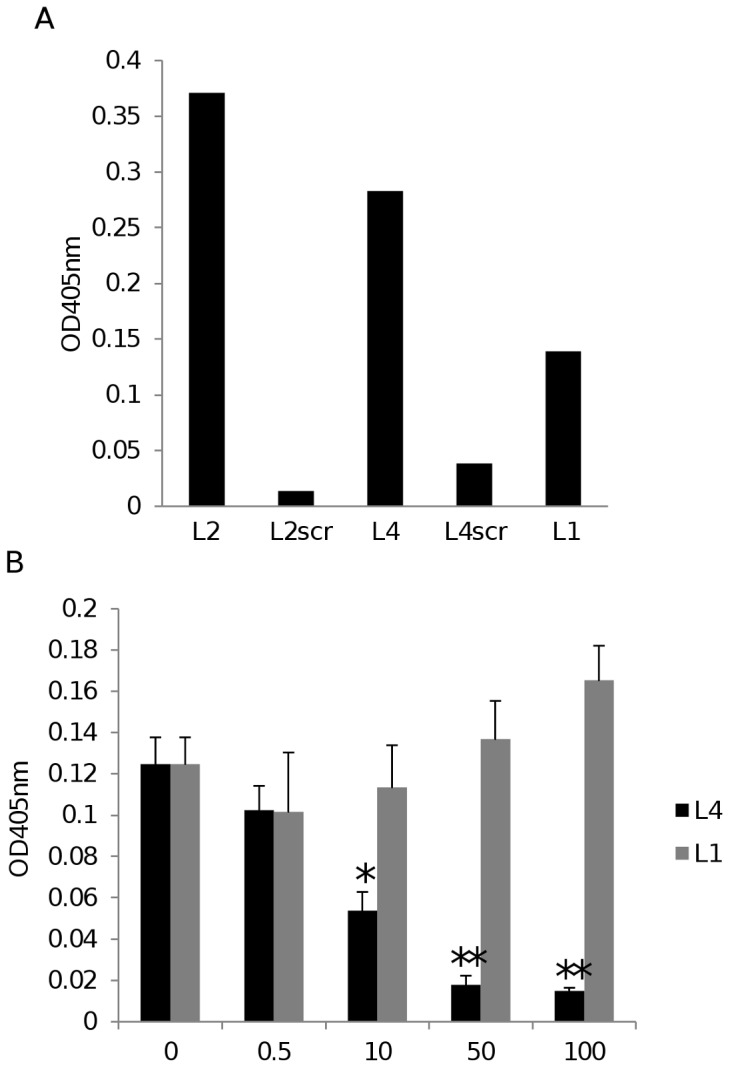
PorA loop 4 and OmpP2 loop 2 peptides bind LamR. (A) Binding of LamR to OmpP2 loop 2 (labeled L2), OmpP2 loop 2scr (L2scr), PorA loop 4 (L4), PorA loop 4scr (L4scr) or PorA loop 1 (L1) coated ELISA plates. Results shown are means of triplicate wells from a representative example from two independent experiments. (B) Binding of DIG-labeled LamR to PorA^20–392^ in the presence of 0.5, 10, 50 or 100 µg of peptide corresponding to PorA loop 4 (L4) or loop 1 (L1). The binding of LamR to PorA^20–392^ in the absence of peptide served as a negative control for inhibition. * *p*<0.05 compared to binding in the absence of peptide. ** *p*<0.01 compared to binding in the absence of peptide. Data shown are means from three independent experiments; in each experiment each sample was tested in triplicate. Error bars indicate SEM.

Further characterization of the PorA and OmpP2 LamR-binding domains was undertaken using synthetic peptides. Several peptides were synthesized (Table 1): one corresponding to the 9-amino acid apical region of OmpP2 loop 2 (residues 91–99); another corresponding to the 34-amino acids of PorA loop 4 (residues 185–218), which we hypothesized was responsible for PorA-LamR binding; and a third corresponding to PorA loop 1 (residues 31–70) for use as a non-LamR binding control peptide. In addition, scrambled peptides based on OmpP2 loop 2 and PorA loop 4 were also synthesized. Each peptide included additional terminal cysteine residues; under oxidizing conditions cysteine sulfhydryl groups spontaneously form disulfide bonds, thus allowing the peptides to circularize and more closely mimic the native loop structures. PorA loop 1 and the two scrambled peptides (PorA loop 4scr and OmpP2 loop 2scr) bound less than either OmpP2 loop 2 or PorA loop 4 to LamR (Fig. 3A). Furthermore, PorA loop 4, but not PorA loop 1 could block the binding of soluble LamR to the solid-phase ligand PorA^20–392^ in a dose-dependent manner (Fig. 3B).

**Table 1:** Synthetic peptides used in this study.

Peptide name	Peptide sequence
OmpP2 loop 2	CASENGSDNFC a
OmpP2 loop 2scr	CSNGFEDNSAC a
PorA loop 4	CPIQNSKSAYTPAYYTKNTNNNLTLVPAVVGKPGSC a
PorA loop 4scr	CNSNGATGKNPPVVTLKKSVYQSNYTAPYANTILPC a
PorA loop 1	CVEGRNYQLQLTEAQAANGGASGQVKVTKVTKAKSRIRTKIC a
L4_1	PIQNSKSAYT
L4_2	AYTPAYYTKN
L4_3	TKNTNNNLTL
L4_4	LTLVPAVVG
L4_5	PAVVGKPGS

a Peptide sequence includes terminal cysteine residues to facilitate disulfide bond formation.

### Loop 4 residues 192–208 are required for optimal LamR-binding

**Figure 4:**
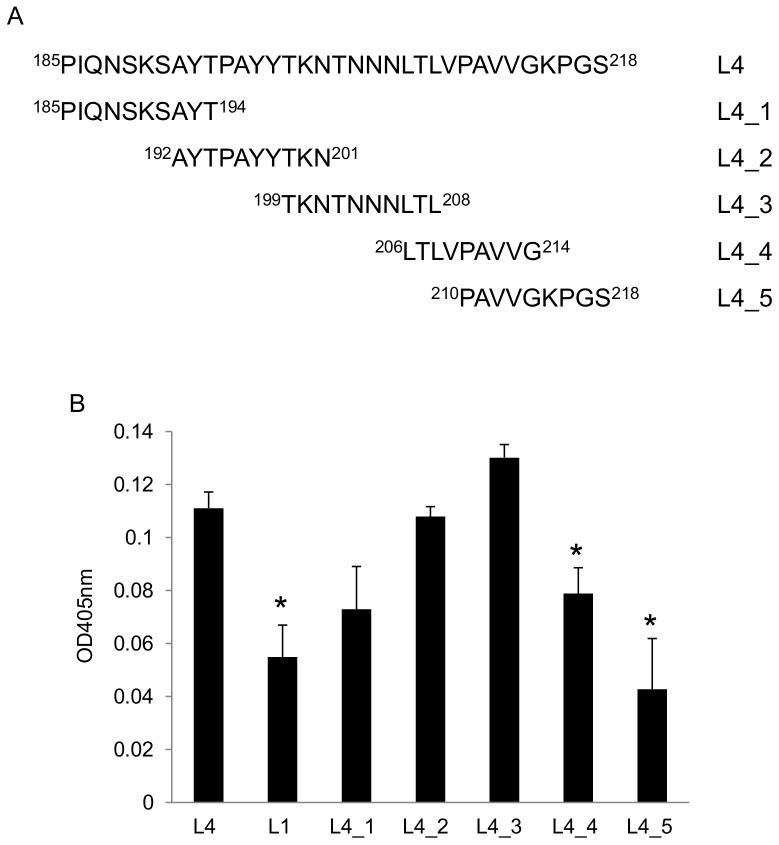
LamR binding activity of PorA loop 4 peptides. (A) Schematic showing the sequence of the overlapping loop 4 peptides (L4_1 to L4_5) and alignment to the MC58 PorA loop 4 sequence (L4). (B) ELISA data showing the binding of LamR to immobilized loop 1 and loop 4 synthetic peptides. Data shown are means from at least three independent experiments; in each experiment each sample was tested in triplicate. * *p*<0.05 compared to binding of LamR to whole loop 4 (L4). Error bars indicate SEM.

Experiments were undertaken to further define the region within the 34-amino acid PorA loop 4 required for optimal LamR binding. Five overlapping peptides (L4_1 to L4_5) were synthesized spanning loop 4 (Table 1 & Fig. 4A). The peptides were immobilized in microtitre wells and DIG-labeled LamR added. After washing, bound LamR was detected and quantified (Fig. 4B). As expected, the control non-LamR binding control peptide (loop 1) showed significantly less binding to LamR compared to whole loop 4. Two of the shorter loop 4 peptides (L4_4 and L4_5) showed significantly reduced LamR-binding compared to whole loop 4 (*p*<0.05), whilst L4_1 showed reduced binding, albeit it not reaching statistical significance (*p* = 0.069). The highest LamR-binding was exhibited by L4_2 and also L4_3, which correspond to the most apical part of loop 4 and together span PorA^192–208^ suggesting that the key residue(s) mediating LamR-binding are localized in this region.

### Deletion of N. meningitidis PorA loop 4 or H. influenzae OmpP2 loop 2 reduces LamR-binding

**Figure 5:**
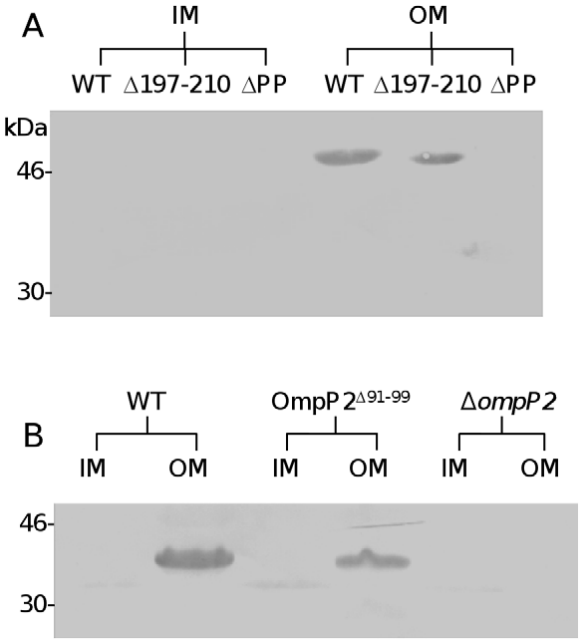
Immunoblots confirm the presence of truncated porins in outer membrane-enriched sub-cellular fractions. Inner and outer membrane fractions (labeled IM and OM, respectively) of MC58 wild-type, MC58PorA^Δ197–210^Δ*pilQ* (labeled Δ197–210) and MC58Δ*porA*Δ*pilQ* double mutant (labeled ΔPP) (A) or *H. influenzae* Rd KW20 wild-type, OmpP2^Δ91–99^ and Δ*ompP2* (B) were probed with anti-PorA monoclonal antibody or rabbit polyclonal anti-OmpP2, respectively.

**Figure 6:**
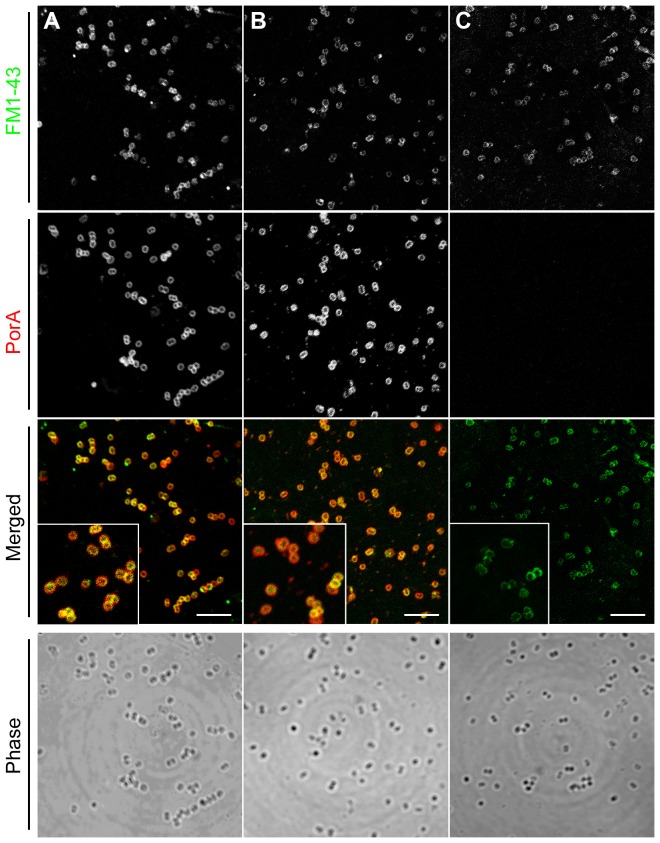
Deletion of loop 4 does not affect insertion of PorA into the meningococcal outer membrane. Immuno-fluorescent images of (A) MC58 wild-type, (B) MC58PorA^Δ197–210^Δ*pilQ* or (C) MC58Δ*porA*Δ*pilQ*. Intact cells of *N. meningitidis* were probed with FM1-43 membrane stain (upper panels) and anti-meningococcal serosubtype P1.7 monoclonal antibody (centre panels). Merged images (lower panels) depict co-localization of FM1-43 (green) and anti-meningococcal serosubtype P1.7 monoclonal antibody (red), in yellow. Phase images (bottom panels) verify the presence of immuno-targets. Insets shown are at 2.5× field magnification. Scale bar: 5 µm.

**Figure 7:**
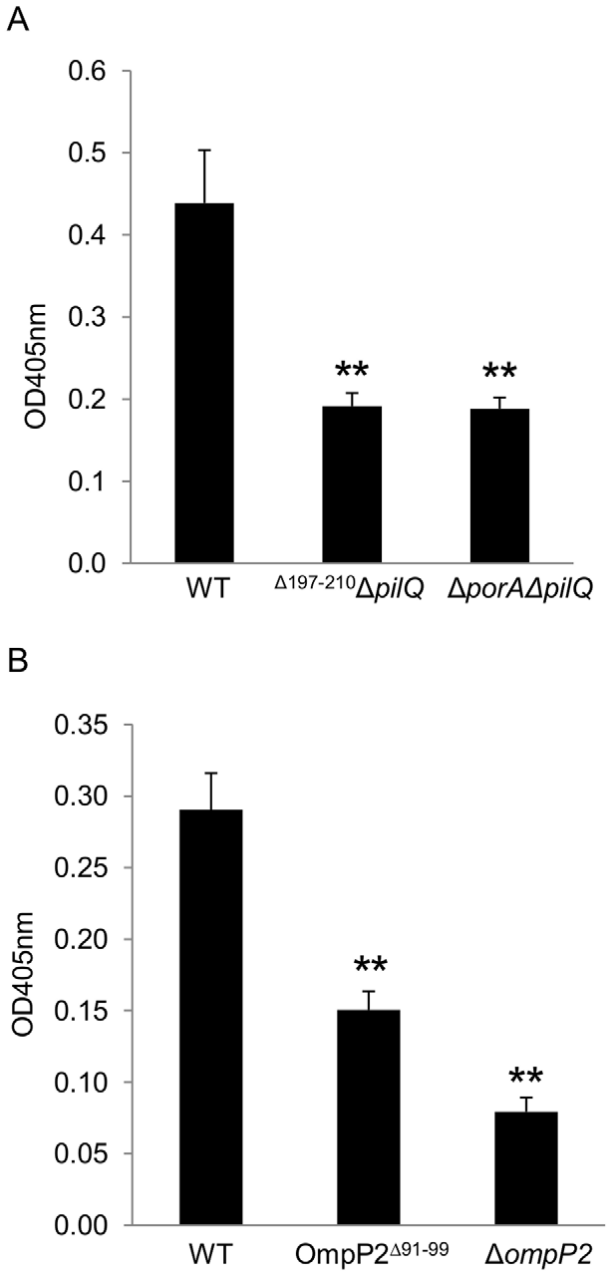
Deletion of PorA loop 4 or OmpP2 loop 2 significantly reduces bacterial LamR-binding. Binding of (A) *N. meningitidis* MC58, MC58PorA^Δ197–210^Δ*pilQ* and MC58Δ*porA*Δ*pilQ* or (B) *H. influenzae* Rd KW20, RdOmpP2^Δ91–99^ or RdΔ*ompP2* to LamR. Specific binding of digoxigenin-labeled bacteria to LamR-coated ELISA plates was determined by subtracting the absorbance in BSA-coated wells from that in LamR-coated wells. Data shown in each panel are means from three independent experiments; in each experiment each sample was tested in triplicate. ** *p*<0.01 compared to wild-type. Error bars indicate SEM.

To determine whether the LamR-binding activity of PorA loop 4 observed using synthetic peptides and recombinant sub-fragments reflect the ability of the protein *in situ* in the bacterial cell to mediate binding to LamR, a derivative of the *N. meningitidis* strain MC58 was constructed in which the central 14 amino acids of PorA loop 4 were deleted (MC58PorA^Δ197–210^). We have previously shown that meningococci have two LamR-binding ligands: PorA and PilQ, and that in the presence of PilQ, deletion of PorA has relatively little effect on observable LamR-binding [22]. Therefore, we introduced a *pilQ* mutation into the MC58PorA^Δ197–210^ genetic background to create MC58PorA^Δ197–210^Δ*pilQ* to enable us to specifically address the effect of loop 4 truncation on PorA-mediated LamR-binding. Truncating loop 4 did not interfere with expression of PorA or the ability of the protein to correctly localize to the meningococcal outer membrane. Immunoblots of cell fractions confirmed the presence of PorA^Δ197–210^ at similar levels to wild-type PorA in meningococcal outer membrane-enriched sub-cellular fractions (Fig. 5A). In addition, a mouse monoclonal antibody directed against loop 1 bound to the surface of strains expressing wild-type PorA or PorA^Δ197–210^ at similar levels as observed by confocal microscopy, but not in strains where *porA* had been inactivated (Fig. 6). LamR-binding exhibited by MC58PorA^Δ197–210^Δ*pilQ* was significantly reduced and similar to the control strain lacking PorA and PilQ (Fig. 7A), thus confirming that the apical region of loop 4 determines PorA-mediated LamR binding by *N. meningitidis* MC58.

Similarly, we constructed a *H. influenzae* Rd KW20 derivative in which the amino acids 91–99 were deleted (RdOmpP2^Δ91–99^). Again, prior to determining the LamR-binding potential of this strain, we first confirmed that truncating loop 2 did not interfere with expression of OmpP2 or the ability of the protein to correctly localize to the *H. influenzae* outer membrane (Fig. 5B). LamR-binding by RdOmpP2^Δ91–99^ was significantly reduced compared to the wild-type (*p*<0.01), albeit the reduction in LamR-binding was not as great as that observed for the control strain lacking OmpP2 (Fig. 7B), perhaps indicating that additional residues of OmpP2 may play a role in optimizing LamR-binding. Control strains with an antibiotic cassette inserted at the *porA* or *ompP2* locus, but no modification to either the *porA* or *ompP2* coding sequence, showed no significant reduction in LamR-binding, thus confirming the direct effect of removing or truncating *porA* or *ompP2* on binding (data not shown).

In summary, we have identified the apical regions of the fourth and second extra-cellular loops of meningococcal MC58 PorA and *H. influenzae* Rd KW20 OmpP2, respectively, as the LamR-binding regions of these bacterial ligands.

## Discussion

Several neurotropic pathogens bind to the 37/67-kDa laminin receptor on host microvascular endothelial cells. In particular, this adherence strategy is used by the bacterial pathogens *S. pneumoniae*, *N. meningitidis* and *H. influenzae* [22]. These species target a common carboxy-terminal recognition site on LamR (amino acids 263–282), since antibodies recognizing this sequence or a peptide corresponding to LamR residues 263–282 could inhibit bacterial binding to microvascular endothelial cells [22]. The bacterial ligands required for LamR-binding were subsequently identified as pneumococcal CbpA, meningococcal PilQ and PorA and *H. influenzae* OmpP2 [22]. Understanding the structural basis for the ability of these ligands to bind LamR could facilitate the design of therapeutic interventions which could prevent or disrupt the interaction and thus engender protection against bacterial meningitis.

To this end, we previously investigated the LamR-binding region of CbpA: a secreted protein that acts as a bridging adhesin to human cells [35]. The CbpA LamR-binding region mapped to a highly conserved surface-exposed loop (residues ^391^EPRNEEK^397^) linking the second and third anti-parallel α-helices of the R2 domain. Pneumococcal strains which failed to bind LamR were shown to have this region missing from CbpA [22]. Two residues within this region were found to be particularly important for LamR binding; pneumococci expressing a CbpA_P392G_-_R393G_ derivative showed significantly reduced binding to recombinant LamR and endothelial cells. CbpA_P392G_-_R393G_-coated microspheres showed significantly reduced binding to mouse cerebral endothelium compared to those bearing wild-type CbpA [22]. Importantly, in a mouse model of pneumococcal meningitis, strains expressing CbpA_P392G_-_R393G_ rarely caused meningitis compared to strains expressing wild-type CbpA, thus underlining the *in vivo* significance of these key residues in the disease process in the mouse model [22].

In this study, we undertook to define the regions of meningococcal PorA and *H. influenzae* OmpP2 required for LamR-binding. Both are abundant outer membrane trimeric porins that contain multiple surface-exposed loop structures, but lack sequences resembling the CbpA LamR-binding domain. Using sub-fragments of recombinant MC58 PorA and Rd KW20 OmpP2, our results suggest that their LamR-binding domains are localized to amino acids 171–240 and amino acids 91–99 respectively, since derivatives containing these regions bound LamR; in contrast derivatives lacking these regions showed significantly reduced LamR-binding. For both PorA and OmpP2, predicted surface-exposed loops mapped to these LamR-binding regions. The importance of these loops was confirmed using synthetic peptides based on their sequences. Peptides corresponding to either PorA loop 4 or OmpP2 loop 2 bound LamR, whilst scrambled peptides based on these loops or a peptide based on PorA loop 1 bound less well. Additionally, PorA loop 4 could significantly inhibit binding of LamR to full-length PorA in contrast to PorA loop 1.

To further refine the region within the 34-amino acid loop 4 required for optimal LamR-interaction, the binding abilities of shorter overlapping peptides spanning the loop were determined. This approach was not undertaken for OmpP2 loop 2, since our data already indicated that optimal binding was dependant on a short 9-amino acid region (residues 91–99) corresponding to the apex of the loop. Likewise for PorA loop 4, the highest LamR-binding was exhibited by short peptides mapping to the loop apex, suggesting that the key residue(s) mediating binding are localized to this region.

LamR-binding exhibited by synthetic peptides or ligand sub-fragments purified under non-native conditions might not accurately reflect the ability of native ligands embedded in the Gram negative outer membrane to interact with LamR. We therefore confirmed our findings using *H. influenzae* and meningococcal LamR-ligands expressed in their *in vivo* context. *H. influenzae* Rd KW20 expressing OmpP2 containing a 9-amino acid deletion from the apex of loop 2 showed significantly reduced LamR-binding compared to wild-type. However, LamR-binding was not reduced to the level determined for the *H. influenzae* Δ*ompP2* control strain, indicating that residues outside of the deleted region may also have a minor effect to modulate binding. Recent investigations into the role of OmpP2 and its loops in pathogenesis have focused on the ability of the protein and some of its loops (principally loop 7) to activate host signaling cascades (including the mitogen-activated protein kinase cascade) and initiate the innate immune response (inducing the release of TNF-alpha and IL-6) in a variety of human cell types [36], [37], [38], [39]. Here we show that, in addition to the demonstrated role of OmpP2 loop 7 in pathogenesis, loop 2 is also biologically relevant as it largely determines the LamR binding activity of OmpP2.

Our analysis of PorA-mediated LamR-binding in the meningococcus was complicated by the presence of PilQ, the second LamR ligand expressed by this organism. Consequently, our LamR-binding experiments were undertaken in *pilQ* knockout derivatives of MC58 to exclude the effects of this protein. Importantly, *N. meningitidis* expressing PorA lacking 14-amino acids from the apex of loop 4 exhibited a significant reduction in LamR-binding compared to strains expressing wild-type PorA. In fact, removing these amino acids from PorA in a *pilQ* knockout background, reduced LamR-binding to levels indistinguishable from that of a *pilQ porA* double knockout mutant. Importantly, this was not due to the inability of the modified PorA to insert into the meningococcal outer membrane. Based on our data, we conclude that the fourteen amino acids of loop 4 represent the sole LamR-binding domain of PorA in this strain. Further deletions or site-directed mutagenesis of residues in this region of PorA may lead to further refinement of this binding domain.

Loop 2 corresponds to variable region 1 of OmpP2 [24]. Thus, antigenic variation at this loop is likely to explain the varying LamR-binding capabilities of *H. influenzae* isolates reported previously [22]. Similarly, the fourteen amino acid LamR-binding region of PorA corresponds to the VR2 of this antigen. Our identification of VR2 as the LamR-binding of PorA in this study is consistent with previous observations from an experiment in which *porA* of a meningococcal isolate with a low LamR-binding activity (Z4682) was replaced with *porA* from MC58 [22]. Strain Z4682 bound LamR at 50% of the level of MC58; following the *porA* replacement, LamR-binding was increased to 94% of MC58 [22]. The PorA variants (VR1, VR2) of MC58 and Z4682, respectively, are: 7, 16-2 and 7.1, 1 (http://pubmlst.org/neisseria/PorA/). Differences in VR1 sequence between variants 7 and 7.1 are minor, involving the presence/absence of a duplication of two residues. In contrast, the differences in amino acid sequence between VR2 variants 16-2 and 1 are considerable (16-2: YYTKNTNNNLTLVP; 1: YVAVENGVAKKVA). Since VR1 corresponds to loop 1, and VR2 to loop 4, this provided circumstantial evidence linking loop 4, but not loop 1 to differences in LamR-binding ability. Here, using a comprehensive approach involving PorA sub-fragments, synthetic peptides and meningococcal strains expressing PorA in which specific extracellular loops were deleted, we confirm that the fourth extra-cellular loop is the LamR-binding region of this protein.

Since over 630 PorA VR2 variants (classified into 20 families) have been identified across meningococcal isolates to date, we suggest that the LamR-binding ability conferred by PorA is variable between strains. In contrast, since PilQ is highly conserved (≥98% identity at amino acid level between genome sequenced meningococcal strains), we hypothesize that PilQ confers a relatively constant LamR-binding capability to strains of different lineages. We are currently determining the LamR-binding domain(s) of PilQ to allow us to investigate this further. Why the meningococcus has evolved to express two LamR-binding ligands, whilst *H. influenzae* and pneumococcus both express only one is unclear. However, meningococci have previously been reported to express multiple adhesins with overlapping ligand-binding capabilities [40]. Functional redundancy is required to maintain colonization because of the propensity of the organism to phase and/or antigenically vary its surface structures. PorA is one such variable surface structure; in addition to being antigenically variable, PorA expression levels vary between isolates and within a population of the same isolate. This is a result of slipped-strand mispairing at a homopolymeric tract in the *porA* promoter [41]. In addition, PorA expression may be absent altogether due to: slipped-strand mispairing at a homopolymeric tract within the *porA* gene [41]; deletion of *porA* [42]; or via the insertion of an IS element [43]. Whether PilQ expression is varied in similar ways and if so, what the genetic mechanisms governing this variation are, are not currently known. However, we suggest that the meningococcus utilizes two LamR-ligands in order to maintain a degree of LamR-binding capability, irrespective of changes in PorA (or possibly PilQ) expression, during the infection process. Since meningococci are normally commensal organisms inhabiting the nasopharynx, and infection of the meninges represents a relatively rare event that is unlikely to contribute to the dissemination of this accidental pathogen, selective pressure leading to such functional redundancy is likely to be applied at mucosal surfaces and not at the meninges.

In summary, we have identified the LamR-binding regions within meningococcal PorA and *H. influenzae* OmpP2. These regions correspond to the apical regions of the fourth and second extra-cellular loops, respectively. Both loops are hyper-variable, suggestive of the molecular basis for the diverse range of LamR-binding capabilities previously reported for meningococcal and *H. influenzae* strains of different lineages. Increased knowledge of the structural motifs of bacterial ligands that interact with LamR may facilitate the design of therapeutic interventions which could disrupt or modulate the interaction of neuroinvasive bacteria with LamR and engender protection against bacterial meningitis. However, given that both OmpP2 loop 2 and PorA loop 4 are hyper-variable, this may hinder the design of strategies to block LamR-binding, which are effective against a wide range of pathogenic strains.

## Materials and Methods

### Bacterial strains and growth conditions

*Escherichia coli* JM109 was used as the host strain for mutagenic constructs and for expression of 6× histidine-tagged recombinant protein fragments (Table S1). This strain was grown at 37°C in Lysogeny Broth (LB) or on LB agar supplemented, where appropriate, with ampicillin, kanamycin or streptomycin and spectinomycin (all at 100 µg ml^−1^). *N. meningitidis* strains were grown at 37°C, in an atmosphere of air plus 5% CO_2_, on Columbia agar with chocolated horse blood (Oxoid) or in Brain Heart Infusion (BHI) broth (Oxoid) supplemented, where appropriate, with streptomycin and spectinomycin (100 µg ml^−1^) and/or kanamycin (50 µg ml^−1^). *H. influenzae* strains were grown at 37°C, in an atmosphere of air plus 5% CO_2_, on Columbia agar with chocolated horse blood (Oxoid) or in BHI broth supplemented with haemin (10 µg ml^−1^) and NAD (10 µg ml^−1^), and where appropriate, with kanamycin (50 µg ml^−1^).

### DNA manipulation

Chromosomal DNA was purified using the DNeasy Tissue kit (Qiagen). Plasmid DNA was prepared by using the QIAprep Spin kit (Qiagen). Restriction enzymes were purchased from Roche and used according to the manufacturer's instructions. DNA sequencing was carried out on an ABI 377 automated DNA sequencer at the School of Biomedical Sciences (University of Nottingham).

### Construction of plasmids encoding PorA sub-fragments

The plasmids encoding PorA^20–392^, PorA^20–170^, PorA^20–258^ and PorA^20–332^ were obtaining by amplifying *porA* fragments from *N. meningitidis* MC58 using a common forward oligonucleotide primer (porA-F1; Table S2) incorporating a BamHI restriction site, and reverse primers porA-R, porA-R1, porA-R2 and porA-R3, respectively, incorporating SalI restriction sites. The resulting amplicons were BamHI/SalI-digested and ligated into BamHI/SalI-digested pQE30 to yield pPorAQE30, pPorA20-170, pPorA20-258 and pPorA20-332, respectively (Table S1). The construct for expression of PorA^95–170^ was obtained by performing inverse PCR using pPorA20-170 as template DNA. The primers used for this (porA-F2 and porA-R4; Table S2) incorporated BglII restriction sites into the amplicon, allowing re-ligation of the product following appropriate enzymatic treatment to yield pPorA95-170. An identical strategy was used to generate pPorA150-258, but utilizing pPorA20-258 as template DNA and primers porA-F3 and porA-R4 (Table S2). Inverse PCR, using pPorAQE30 as template, was utilized to obtain PorA^Δ58–94^, PorA^Δ125–149^ and PorA^Δ169–240^. The primers pairs used for this: porA-F4/-R5 (PorA^Δ58–94^), porA-F3/-R6 (PorA^Δ125–149^) and porA-F5/-R7 (PorA^Δ169–240^) incorporated BglII restriction sites into the amplicon, allowing re-ligation of the product following BglII treatment to yield pPorA^Δ58–94^, pPorA^Δ125–149^ and pPorA^Δ169–240^, respectively (Table S1).

### Construction of plasmids encoding OmpP2 sub-fragments

The plasmid encoding OmpP2^24–359^ was obtaining by amplifying *ompP2* from *H. influenzae* Rd KW20 using primers P2F1 (incorporating a BamHI restriction site; Table S2) and P2R1 (incorporating a SalI restriction site). The resulting amplicon was BamHI/SalI-digested and ligated into BamHI/SalI-digested pQE30 to yield pNJO74 (Table S1). The plasmid encoding OmpP2^224–359^ was constructed by inverse PCR using pNJO74 as template and P2Δ1-4I_F and P2Δ1-4I_R primers (Table S2). The amplified product was BamHI-digested and then re-ligated to yield pMSA1 (Table S1). The plasmid encoding OmpP2^24–225^ was also constructed by IPCR using pNJO74 as template, but utilizing P2Δ5-8I_F and P2Δ5-8I_R primers (Table S2). The amplified product was SalI-digested and then re-ligated to yield pMSA2 (Table S1). Inverse PCR using pNJO74 as template was also utilized to derive the plasmids encoding OmpP2 lacking loop 1, 2, 3 or 4. Primer pairs utilized were: P2ΔL1I_F and P2ΔL1I_R (loop 1 deletion), P2ΔL2I_F and P2ΔL2I_R (loop 2 deletion), P2ΔL3I_F and P2ΔL3I_R (loop 3 deletion) and P2ΔL4I_F and P2ΔL4I_R (loop 4 deletion; Table S2). These primers incorporated BglII restriction sites into the amplicons, allowing re-ligation of the products following appropriate enzymatic treatment to yield pMSA3, pMSA4, pMSA5 and pMSA6, respectively (Table S1).

### Protein expression and purification

Recombinant histidine-tagged LamR was purified as described previously [22]. For the expression of PorA and OmpP2 derivatives, plasmids were transformed into *E. coli* JM109, cultures grown to exponential phase (OD_600_>0.5) and induced with isopropyl β-D-1-thiogalactopyranoside (IPTG; 1 mM) for 3 h. Recombinant proteins were then affinity-purified using HisPur™ Cobalt Resin (Pierce) under denaturing conditions using a previously described method [44]. Final column elutes were buffer exchanged into 1× PBS using PD-10 desalting columns (GE Healthcare) according to the manufacturer's instructions.

### SDS-PAGE and immunoblotting

Proteins were electrophoretically separated using polyacrylamide gels (Mini-Protean III; Bio-Rad). Proteins were stained using SimplyBlue Safestain™ (Invitrogen) or transferred to nitrocellulose membranes. Membranes were probed with either mouse anti-pentahistidine antibody (Qiagen; 1∶5000 diluted) or mouse monoclonal anti-PorA (NIBSC code: 01/514; 1∶5000 diluted) or rabbit polyclonal anti-OmpP2 (1∶5000 diluted) in blocking buffer (5% [wt/vol] non fat dry milk, 0.1% [vol/vol] Tween 20 in 1× phosphate-buffered saline [PBS]) and incubated for 2 h. After being washed in PBS with 0.1% Tween 20, membranes were incubated for 2 h with 1∶30,000-diluted goat anti-mouse (or anti-rabbit) IgG-alkaline phosphatase conjugate (Sigma) and washed with PBS with 0.1% Tween 20. Immunoblots were developed using BCIP/NBT-Blue liquid substrate (Sigma).

### DIG-labeling of proteins and bacteria

DIG-labeling was carried using the Roche DIG-NHS Protein labeling kit, used as per manufacturer's instructions. Briefly, for protein the pH was adjusted to pH 9.0 by addition of 2 M sodium carbonate, and DIG-NHS reagent added in a 1∶10 molar ratio. Reactions were incubated at room temperature for 1 h, and then unbound DIG-NHS was removed using PD-10 desalting columns (GE Healthcare). For bacteria, cells were harvested from an overnight plate, washed 3 times in PBS with 0.05% Tween 20 (PBST) and resuspended in sodium carbonate buffer (150 mM; 142 mM NaHCO_3_, 8 mM Na_2_SO_3_, pH 9.0). Bacteria (optical density at 600 nm = 1.0) were then labeled with 10 µg ml^−1^ DIG-NHS at room temperature for 30 min. Bacteria were then washed 3 times with PBST and resuspended in PBS containing 1% bovine serum albumin (BSA; Sigma).

### Enzyme-linked immunosorbent assays

100 µl aliquots of 5 µg ml^−1^ protein in sodium carbonate buffer were added to a CovaLink NH microplate (Nalge Nunc International, USA), and incubated at room temperature for 1 h or overnight at 4°C. The plate was washed three times with PBST and blocked with 1% BSA/PBS for 1 h. After removal of the blocking solution, 100 µl of 5 µg ml^−1^ DIG-labeled protein or DIG-labeled bacteria (OD_600_ at 0.04) was added and incubated at room temperature for 90 min or at 4°C overnight. Following vigorous washing in PBST (5 washes with 5 min soaking times), 100 µl of anti-DIG HRP conjugate (Roche), diluted 1∶5000 in 1% BSA/PBS, was added to wells and incubated for 1 h. Plates were again vigorously washed and color developed by adding 100 µl ABTS substrate (Roche). Plates were read with an ELISA reader (Biotek EL800) at an absorbance of 405 nm. Alternatively, for unlabelled LamR, 100 µl of protein (5 µg ml^−1^) was added to coated wells. After washing, bound LamR was detected using rabbit polyclonal anti-LamR (diluted 1∶1000) in 1% BSA/PBS followed by goat anti-rabbit IgG-HRP conjugate (Sigma; diluted 1∶10,000). Inhibition assays were performed as described above, except that 0.5 µg aliquots of DIG-labeled LamR were pre-incubated with 0.5–100 µg of peptide for 4 h at room temperature prior to being added to coated wells. OD values shown are minus OD values obtained from control wells coated with 100 µl aliquots of 1% BSA. Statistical significance was determined using two-tailed Student's *t* test.

### Synthetic peptides

The synthetic PorA and OmpP2 peptides utilized in this study are detailed in Table 1. All were synthesized (high purity grade; peptide purity >95%) by GenScript, USA.

### PorA loop 4 truncation in N. meningitidis

A 42-bp deletion in the coding sequence of MC58 *porA* was introduced into a previously constructed plasmid, pPorA-4d (Table S1), by inverse PCR using primers L4-F1 and L4-R1 (Table S2). Amplification resulted in a product containing the desired deletion and incorporating unique BglII and BamHI sites, thus allowing re-ligation of the fragment to form pPorAΔL4 (Table S1). Inverse PCR using primers L4-F2 and L4-R2 was then used to introduce a BglII site downstream of *porA*, into which a BglII-digested Ω cassette (encoding resistance to spectinomycin and streptomycin) [45] was inserted to form pPorAΔL4Ω (Table S1). This plasmid was subsequently used to mutate *N. meningitidis* MC58 by natural transformation and allelic exchange as previously described [46]. The *porA* loop 4 deletion in the resulting mutant (MC58PorA^Δ197–210^) was confirmed by PCR analysis and DNA sequencing. Growth curve assays carried out using liquid cultures showed no significant differences between MC58PorA^Δ197–210^ and the wild-type strain (data not shown). The *pilQ* knockout mutation was subsequently introduced into this strain to generate MC58PorA^Δ197–210^Δ*pilQ* using previously described methods [22].

### OmpP2 loop 2 truncation in H. influenzae

A 2.7-kb region containing the *ompP2* gene and flanking DNA was amplified from *H. influenzae* Rd KW20 using primers F1 and mP2_R (Table S2) and TA-cloned into pGEM-T Easy to yield pMSA8 (Table S1). This template was subject to inverse PCR using OmgF and OmgR primers, thus incorporating BamHI restriction sites into the amplicon and a copy of a DNA uptake sequence (5′-AAGTGCGGTCA-3′) which is required for efficient DNA uptake by *H. influenzae* [47]. The BamHI site was used to introduce a BamHI-digested kanamycin resistance cassette downstream of, and in the same orientation as *ompP2*, to generate pMSA15. Inverse PCR utilizing primers P2ΔL2I_F and P2ΔL2I_R was subsequently used to remove the nucleotides encoding OmpP2 amino acids 91–99. Use of P2ΔL2I_F and P2ΔL2I_R incorporated BglII-sites into the amplicon, thus allowing re-ligation to form pMSA16. Nucleotide sequencing revealed a single base pair deletion present in the 5′ end of the *ompP2* coding sequence in pMSA8 and in subsequent constructs which would lead to premature translation of *ompP2* in *H. influenzae*. To repair this, a 539-bp mega primer spanning the mutated region was amplified from Rd KW20 chromosomal DNA using primers F1 and MegaR (Table S2). The mega primer was then used in conjunction with primer mP2_R using pMSA16 as template to generate a linear repaired mutagenic product comprising of upstream flanking sequence, *ompP2* lacking nucleotides encoding loop 2, kanamycin resistance cassette and downstream flanking sequence. This product was used to naturally transform *H. influenzae* Rd KW20 using a previously described method for the natural transformation of *N. meningitidis* [46]. The *ompP2* loop 2 deletion in the resulting mutant (RdOmpP2^Δ91–99^) was confirmed by PCR analysis and DNA sequencing. Growth curve assays carried out using liquid cultures showed no significant differences between RdOmpP2^Δ91–99^ and the wild-type strain (data not shown).

### Mutation of OmpP2 in H. influenzae

796-bp of *ompP2* was removed from pMSA15 by inverse PCR using primers ΔP2F and P2ΔL2I_R. These primers incorporated BglII-sites allowing re-ligation of the amplicon to form pMSA17 (Table S1). This PCR template was then used in conjunction with primers F1 and P2FR to yield a linear mutagenic PCR product that was used to naturally transform *H. influenzae* Rd KW20 using a previously described method [46]. The *ompP2* deletion in the resulting mutant (RdΔ*ompP2*) was confirmed by PCR analysis and DNA sequencing. Growth curve assays carried out using liquid cultures showed no significant differences between RdΔ*ompP2* and the parental strain (data not shown).

### Cell fractionation and confocal microscopy

Cell fractionation was undertaken as described previously [48]. For confocal microscopy, growth from overnight cultures was harvested by centrifugation (4000× *g* for 10 min), resuspended in 1× PBS, and the OD_600_ adjusted to 1.0. 50 µl aliquots were then added to Knittel adhesive glass slides (SLS) and left at room temperature for 1 h. Bacteria were fixed using 4% paraformaldehyde for 10 min. Slides were washed four times in 1× PBS and then blocked with 1% BSA/PBS for 1 h. Surface-accessible PorA was labeled with mouse anti-meningococcal serosubtype P1.7 monoclonal primary antibody (1∶25 diluted; NIBSC code: 01/514) for 1 h. Following washing in 1× PBS, goat anti-mouse IgG-cascade blue conjugate (1∶800 diluted; Invitrogen) was added for 1 h. Following further washes in 1× PBS, membranes were stained with FM1-43 (10 µM; Invitrogen) in Hank's Balanced Salt Solution (Invitrogen) for 30 min in the dark. Following a final 1× PBS wash, coverslips were mounted with Prolong Gold anti-fade (Invitrogen) and images acquired on a Zeiss LSM700 confocal microscope using a 63× objective. Images are single sections (300 nm) and data was collected from different fluorophores in separate channels. Images were processed using Image J and Adobe Photoshop.

## Supporting Information

Table S1 **Bacterial strains and plasmids.**
(DOCX)

Table S2 **List of primers used in this study.**
(DOCX)

## References

[1] Pollard AJ, Perret KP, Beverley PC (2009) Maintaining protection against invasive bacteria with protein-polysaccharide conjugate vaccines. Nat Rev Immunol 9: 213–220. 19214194 10.1038/nri2494

[2] Panatto D, Amicizia D, Lai PL, Gasparini R (2011) *Neisseria meningitidis* B vaccines. Expert Rev Vaccines 10: 1337–1351. 21919622 10.1586/erv.11.103

[3] Nelson J, McFerran N, Pivato G, Chambers E, Doherty C, et al (2008) The 67 kDa laminin receptor: structure, function and role in disease. Biosci Rep 28: 33–48. 18269348 10.1042/BSR20070004

[4] Landowski T, Dratz E, Starkey J (1995) Studies of the structure of the metastasis-associated 67 kDa laminin binding protein: fatty acid acylation and evidence supporting dimerization of the 32 kDa gene product to form the mature protein. Biochemistry 5: 11276–11287. 10.1021/bi00035a037 7669786

[5] Butò S, Tagliabue E, Ardini E, Magnifico A, Ghirelli C, et al (1998) Formation of the 67-kDa laminin receptor by acylation of the precursor. J Cell Biochem 69: 244–251. 9581863 10.1002/(sici)1097-4644(19980601)69:3<244::aid-jcb2>3.0.co;2-r

[6] Hundt C, Peyrin JM, Haik S, Gauczynski S, Leucht C, et al (2001) Identification of interaction domains of the prion protein with its 37-kDa/67-kDa laminin receptor. EMBO J 20: 5876–5886. 11689428 10.1093/emboj/20.21.5876 PMC125289

[7] Rao NC, Barsky SH, Terranova VP, Liotta LA (1983) Isolation of a tumor cell laminin receptor. Biochem Biophys Res Commun 111: 804–808. 6301485 10.1016/0006-291x(83)91370-0

[8] Malinoff HL, Wicha MS (1983) Isolation of a cell surface receptor protein for laminin from murine fibrosarcoma cells. J Cell Biol 96: 1475–1479. 6302102 10.1083/jcb.96.5.1475 PMC2112662

[9] Lesot H, Kühl U, Mark K (1983) Isolation of a laminin-binding protein from muscle cell membranes. EMBO J 2: 861–865. 16453457 10.1002/j.1460-2075.1983.tb01514.x PMC555201

[10] Menard S, Castronovo V, Tagliabue E, Sobel ME (1997) New insights into the metastasis-associated 67 kD laminin receptor. J Cell Biochem 67: 155–165. 9328821

[11] Givant-Horwitz V, Davidson B, Reich R (2004) Laminin-induced signaling in tumor cells. Cancer Res 64: 3572–3579. 15150114 10.1158/0008-5472.CAN-03-3424

[12] Scheiman J, Tseng J-C, Zheng Y, Meruelo D (2009) Multiple functions of the 37/67-kDa laminin receptor make it a suitable target for novel cancer gene therapy. Mol Ther 18: 63–74. 19724263 10.1038/mt.2009.199 PMC2839218

[13] Scheiman J, Jamieson KV, Ziello J, Tseng J-C, Meruelo D (2010) Extraribosomal functions associated with the C terminus of the 37/67-kDa laminin receptor are required for maintaining cell viability. Cell Death and Dis 1: e42. 10.1038/cddis.2010.19 PMC3019570 21243100

[14] Kinoshita K, Kaneda Y, Sato M, Saeki Y, Wataya-Kaneda M, et al (1998) LBP-p40 binds DNA tightly through associations with histones H2A, H2B, and H4. Biochem Biophys Res Commun 253: 277–282. 9878528 10.1006/bbrc.1998.9699

[15] Wang KS, Kuhn RJ, Strauss EG, Ou S, Strauss JH (1992) High-affinity laminin receptor is a receptor for Sindbis virus in mammalian cells. J Virol 66: 4992–5001. 1385835 10.1128/jvi.66.8.4992-5001.1992 PMC241351

[16] Thepparit C, Smith DR (2004) Serotype-specific entry of dengue virus into liver cells: identification of the 37-kilodalton/67-kilodalton high-affinity laminin receptor as a dengue virus serotype 1 receptor. J Virol 78: 12647–12656. 15507651 10.1128/JVI.78.22.12647-12656.2004 PMC525075

[17] Akache B, Grimm D, Pandey K, Yant SR, Xu H, et al (2006) The 37/67-kilodalton laminin receptor is a receptor for adeno-associated virus serotypes 8, 2, 3, and 9. J Virol 80: 9831–9836. 16973587 10.1128/JVI.00878-06 PMC1617255

[18] Protopopova E, Sorokin A, Konovalova S, Kachko A, Netesov S, et al (1999) Human laminin binding protein as a cell receptor for tick-borne encephalitis virus. Zentralblatt fur Bacteriologie 289: 632–638.

[19] Ludwig GV, Kondig JP, Smith JF (1996) A putative receptor for Venezuelan equine encephalitis virus from mosquito cells. J Virol 70: 5592–5599. 8764073 10.1128/jvi.70.8.5592-5599.1996 PMC190519

[20] McNichol BA, Rasmussen SB, Carvalho HM, Meysick KC, O'Brien AD (2007) Two domains of cytotoxic necrotizing factor type 1 bind the cellular receptor, laminin receptor precursor protein. Infect Immun 75: 5095–5104. 17709415 10.1128/IAI.00075-07 PMC2168285

[21] Gauczynski S, Peyrin JM, Haik S, Leucht C, Hundt C, et al (2001) The 37-kDa/67-kDa laminin receptor acts as the cell-surface receptor for the cellular prion protein. EMBO J 20: 5863–5875. 11689427 10.1093/emboj/20.21.5863 PMC125290

[22] Orihuela CJ, Mahdavi J, Thornton J, Mann B, Wooldridge KG, et al (2009) Laminin receptor initiates bacterial contact with the blood brain barrier in experimental meningitis models. J Clin Invest 119: 1638–1646. 19436113 10.1172/JCI36759 PMC2689107

[23] van der Ley P, Heckels JE, Virji M, Hoogerhout P, Poolman JT (1991) Topology of outer membrane porins in pathogenic *Neisseria spp* . Infect Immun 59: 2963–2971. 1652557 10.1128/iai.59.9.2963-2971.1991 PMC258120

[24] Srikumar R, Dahan D, Gras MF, Ratcliffe MJ, van Alphen L, et al (1992) Antigenic sites on porin of *Haemophilus influenzae* type b: mapping with synthetic peptides and evaluation of structure predictions. J Bacteriol 174: 4007–4016. 1375930 10.1128/jb.174.12.4007-4016.1992 PMC206110

[25] Sikkema DJ, Murphy TF (1992) Molecular analysis of the P2 porin protein of non-typeable *Haemophilus influenzae* . Infect Immun 60: 5204–5211. 1280627 10.1128/iai.60.12.5204-5211.1992 PMC258298

[26] Bell J, Grass S, Jeanteur D, Munson RS (1994) Diversity of the P2 protein among nontypeable *Haemophilus influenzae* isolates. Infect Immun 62: 2639–2643. 8188390 10.1128/iai.62.6.2639-2643.1994 PMC186559

[27] McGuinness B, Barlow AK, Clarke IN, Farley JE, Anilionis A, et al (1990) Deduced amino acid sequences of class 1 protein (PorA) from three strains of *Neisseria meningitidis*. Synthetic peptides define the epitopes responsible for serosubtype specificity. J Exp Med 171: 1871–1882. 1693651 10.1084/jem.171.6.1871 PMC2187959

[28] Maiden MCJ, Suker J, McKenna AJ, Bygraves JA, Feavers IM (1991) Comparison of the class 1 outer membrane proteins of eight serological reference strains of *Neisseria meningitidis* . Mol Microbiol 5: 727–736. 1904526 10.1111/j.1365-2958.1991.tb00743.x

[29] Frasch CE, Zollinger WD, Poolman JT (1985) Serotype antigens of *Neisseria meningitidis* and a proposed scheme for designation of serotypes. Rev Infect Dis 7: 504–510. 2412271 10.1093/clinids/7.4.504

[30] Jolley K, Brehony C, Maiden M (2007) Molecular typing of meningococci: recommendations for target choice and nomenclature. FEMS Microbiol Rev 31: 89–96. 17168996 10.1111/j.1574-6976.2006.00057.x

[31] de Filippis I, Gopalan V, Huyen Y (2011) PorA VR3 Typing Database: A web-based resource for the determination of PorA VR3 alleles of *Neisseria meningitidis* . Infect Genet Evol 11: 248–249. 20801234 10.1016/j.meegid.2010.08.011

[32] Poolman JT, Bakaletz L, Cripps A, Denoel PA, Forsgren A, et al (2000) Developing a nontypeable *Haemophilus influenzae* (NTHi) vaccine. Vaccine 19, Suppl 1: S108–S115. 11163473 10.1016/s0264-410x(00)00288-7

[33] Granoff DM (2010) Review of meningococcal group B vaccines. Clin Infect Dis 50 Suppl 2: S54–65. 20144017 10.1086/648966 PMC2820413

[34] Yi K, Murphy TF (1997) Importance of an immunodominant surface-exposed loop on outer membrane protein P2 of nontypeable *Haemophilus influenzae* . Infect Immun 65: 150–155. 8975905 10.1128/iai.65.1.150-155.1997 PMC174569

[35] Hakenbeck R, Madhour A, Denapaite D, Brückner R (2009) Versatility of choline metabolism and choline-binding proteins in *Streptococcus pneumoniae* and commensal streptococci. FEMS Microbiol Rev 33: 572–586. 19396958 10.1111/j.1574-6976.2009.00172.x

[36] Galdiero S, Capasso D, Vitiello M, D'Isanto M, Pedone C, et al (2003) Role of surface-exposed loops of *Haemophilus influenzae* protein P2 in the mitogen-activated protein kinase cascade. Infect Immun 71: 2798–2809. 12704154 10.1128/IAI.71.5.2798-2809.2003 PMC153271

[37] Galdiero S, Vitiello M, Amodeo P, D'Isanto M, Cantisani M, et al (2006) Structural requirements for proinflammatory activity of porin P2 Loop 7 from *Haemophilus influenzae* . Biochemistry 45: 4491–4501. 16584185 10.1021/bi052262p

[38] Severino V, Chambery A, Vitiello M, Cantisani M, Galdiero S, et al (2009) Proteomic analysis of human U937 cell line activation mediated by *Haemophilus influenzae* type b P2 porin and its surface-exposed loop 7. J Proteome Res 9: 1050–1062. 10.1021/pr900931n 20043682

[39] Vitiello M, Finamore E, Cantisani M, Bevilacqua P, Incoronato N, et al (2011) P2 porin and loop L7 from *Haemophilus influenzae* modulate expression of IL-6 and adhesion molecules in astrocytes. Microbiol Immunol 55: 347–356. 21288261 10.1111/j.1348-0421.2011.00318.x

[40] Virji M (2009) Pathogenic neisseriae: surface modulation, pathogenesis and infection control. Nature 7: 274–286. 10.1038/nrmicro2097 19287450

[41] van der Ende A, Hopman CTP, Dankert J (2000) Multiple mechanisms of phase variation of PorA in *Neisseria meningitidis* . Infect Immun 68: 6685–6690. 11083782 10.1128/iai.68.12.6685-6690.2000 PMC97767

[42] van der Ende A, Hopman CT, Dankert J (1999) Deletion of *porA* by recombination between clusters of repetitive extragenic palindromic sequences in *Neisseria meningitidis* . Infect Immun 67: 2928–2934. 10338501 10.1128/iai.67.6.2928-2934.1999 PMC96602

[43] Newcombe J, Cartwright K, Dyer S, McFadden J (1998) Naturally occurring insertional inactivation of the *porA* gene of *Neisseria meningitidis* by integration of IS*1301* . Mol Microbiol 30: 453–454. 9791188 10.1046/j.1365-2958.1998.01056.x

[44] Tunio SA, Oldfield NJ, Ala'Aldeen DAA, Wooldridge KG, Turner DP (2010) The role of glyceraldehyde 3-phosphate dehydrogenase (GapA-1) in *Neisseria meningitidis* adherence to human cells. BMC Microbiol 10: 280. 21062461 10.1186/1471-2180-10-280 PMC2994834

[45] Prentki P, Krisch HM (1984) *In vitro* insertional mutagenesis with a selectable DNA fragment. Gene 29: 303–313. 6237955 10.1016/0378-1119(84)90059-3

[46] Hadi HA, Wooldridge KG, Robinson K, Ala'Aldeen DA (2001) Identification and characterization of App: an immunogenic autotransporter protein of *Neisseria meningitidis* . Mol Microbiol 41: 611–623. 11532129 10.1046/j.1365-2958.2001.02516.x

[47] Danner DB, Deich RA, Sisco KL, Smith HO (1980) An eleven-base-pair sequence determines the specificity of DNA uptake in *Haemophilus* transformation. Gene 11: 311–318. 6260577 10.1016/0378-1119(80)90071-2

[48] Tunio SA, Oldfield NJ, Berry A, Ala'Aldeen DAA, Wooldridge KG, et al (2010) The moonlighting protein fructose-1, 6-bisphosphate aldolase of *Neisseria meningitidis*: surface localization and role in host cell adhesion. Mol Microbiol 76: 605–615. 20199602 10.1111/j.1365-2958.2010.07098.x

