# Supplementary material for: Mapping the Laminin Receptor Binding Domains of Neisseria meningitidis PorA and Haemophilus influenzae OmpP2
Source: PLoS One. 2012 Sep 25;7(9):e46233. doi: 10.1371/journal.pone.0046233 (PMC3457995; doi:10.1371/journal.pone.0046233)
Supplement: Table S1 — Bacterial strains and plasmids. (DOCX) [file pone.0046233.s001.docx]

**TABLE S1. Bacterial strains and plasmids**

| Strain or plasmid | Description | Source or reference |
| --- | --- | --- |
| *E. coli* JM109 | *end*A1 *rec*A1 *gyr*A96 *thi hsd*R17 (r_K_^-^r_K_^-^) *rel*A1 *sup*E44 Δ(*lac*-*pro*AB) [F′ *tra*D36 *pro*AB *laq*I^q^ZΔM15] | Promega |
| *N. meningitidis* MC58 | wild-type serogroup B strain | [1] |
| *N. meningitidis* MC58PorA^Δ197-210^ | MC58 derivative expressing PorA lacking 14-residues of loop 4; streptomycin/spectinomycin resistant | This study |
| *N. meningitidis* MC58Δ*pilQ* | *pilQ* mutant derivative of MC58; kanamycin resistant | [2] |
| *N. meningitidis* MC58PorA^Δ197-210^Δ*pilQ* | *pilQ* mutant derivative of MC58PorA^Δ197-210^ | This study |
| *N. meningitidis* MC58Δ*porA*Δ*pilQ* | *porA* and *pilQ* mutant derivative of MC58 | [2] |
| *H. influenzae* Rd KW20 | wild-type nontypeable strain | [3] |
| *H. influenzae* RdOmpP2^Δ91-99^ | Rd KW20 expressing OmpP2 lacking 9-residues of loop 2; kanamycin resistant | This study |
| *H. influenzae* RdΔ*ompP2* | *ompP2* mutant derivative of Rd KW20; kanamycin resistant | This study |
| pQE30 | Expression vector encoding resistance to ampicillin | Qiagen |
| pPorAQE30 | pQE30-based plasmid encoding PorA^20-392^ | This study |
| pPorA20-170 | pQE30-based plasmid encoding PorA^20-170^ | This study |
| pPorA20-258 | pQE30-based plasmid encoding PorA^20-258^ | This study |
| pPorA20-332 | pQE30-based plasmid encoding PorA^20-332^ | This study |
| pPorA95-170 | pQE30-based plasmid encoding PorA^95-170^ | This study |
| pPorA150-258 | pQE30-based plasmid encoding PorA^150-258^ | This study |
| pPorA^Δ58-94^ | pQE30-based plasmid encoding PorA^Δ58-94^ | This study |
| pPorA^Δ125-149^ | pQE30-based plasmid encoding PorA^Δ125-149^ | This study |
| pPorA^Δ169-240^ | pQE30-based plasmid encoding PorA^Δ169-240^ | This study |
| pPorA-4d | MC58 *porA* and flanking regions cloned in pGEM-T Easy | [2] |
| pPorAΔL4 | Derivative of pPorA-4d incorporating a 42bp deletion of *porA* | This study |
| pPorAΔL4Ω | pPorAΔL4-derivative containing streptomycin/spectinomycin resistance cassette downstream of *porA* | This study |
| pNJO74 | pQE30-based plasmid encoding OmpP2^24-359^ | This study |
| pMSA1 | pQE30-based plasmid encoding OmpP2^224-359^ | This study |
| pMSA2 | pQE30-based plasmid encoding OmpP2^24-225^ | This study |
| pMSA3 | pQE30-based plasmid encoding OmpP2^Δ45-61^ | This study |
| pMSA4 | pQE30-based plasmid encoding OmpP2^Δ91-99^ | This study |
| pMSA5 | pQE30-based plasmid encoding OmpP2^Δ125-151^ | This study |
| pMSA6 | pQE30-based plasmid encoding OmpP2^Δ177-195^ | This study |
| pGEM-T Easy | Cloning vector encoding resistance to ampicillin | Promega |
| pMSA8 | pGEM-T Easy plasmid containing *ompP2* and flanking sequence | This study |
| pJMK30 | Source of the kanamycin resistance cassette | [4] |
| pMSA15 | pMSA8-derivative containing a kanamycin cassette downstream of *ompP2* | This study |
| pMSA16 | pMSA15-derivative encoding OmpP2^Δ91-99^ | This study |
| pMSA17 | pMSA15-derivative containing a 796-bp deletion in *ompP2* | This study |

References

1. Tettelin H, Saunders NJ, Heidelberg J, Jeffries AC, Nelson KE, et al. (2000) Complete genome sequence of *Neisseria meningitidis* serogroup B strain MC58. Science 287: 1809-1815.

2. Orihuela CJ, Mahdavi J, Thornton J, Mann B, Wooldridge KG, et al. (2009) Laminin receptor initiates bacterial contact with the blood brain barrier in experimental meningitis models. J Clin Invest 119: 1638-1646.

3. Fleischmann RD, Adams MD, White O, Clayton RA, Kirkness EF, et al. (1995) Whole-genome random sequencing and assembly of *Haemophilus influenzae* Rd. Science 269: 496-512.

4. van Vliet AH, Wooldridge KG, Ketley JM (1998) Iron-responsive gene regulation in a *Campylobacter jejuni fur* mutant. J Bacteriol 180: 5291-5298.
