# Supplementary material for: Mapping the Laminin Receptor Binding Domains of Neisseria meningitidis PorA and Haemophilus influenzae OmpP2
Source: PLoS One. 2012 Sep 25;7(9):e46233. doi: 10.1371/journal.pone.0046233 (PMC3457995; doi:10.1371/journal.pone.0046233)
Supplement: Table S2 — List of primers used in this study. (DOCX) [file pone.0046233.s002.docx]

**TABLE S2. List of primers used in this study**

| Primer | DNA sequence *^a^* | Restriction site |
| --- | --- | --- |
| porA-F1 | CGCGGATCCGATGTCAGCCTATACGGCGAAATC | BamHI |
| porA-R | ACGCGTCGACTTAGAATTTGTGGCGCAAACCGACG | SalI |
| porA-R1 | ACGCGTCGACATCGTAGCGTACGGAAACCG | SalI |
| porA-R2 | ACGCGTCGACGCTGCCGATCAAGAACAA | SalI |
| porA-R3 | ACGCGTCGACGTCGAAACCATGGGCATAGC | SalI |
| porA-F2 | GCGAGATCTCTTGAGCAAGACGTATCCGTTG | BglII |
| porA-R4 | GCGAGATCTGCCGTATAGGCTGACATC | BglII |
| porA-F3 | GCGAGATCTGTGGCTTCGCAATTGGGTATT | BglII |
| porA-F4 | GCGAGATCTGCTTGAGCAAGACGTATCCG | BglII |
| porA-R5 | GGCAGATCTGTAACTTTTACCTGACCGCTCG | BglII |
| porA-R6 | GCGAGATCTCGTACCGAATTCGCCTGC | BglII |
| porA-F5 | GCGAGATCTGCGAGACACGCCAATGTCG | BglII |
| porA-R7 | CGCAGATCTGCGTACGGAAACCGGCATGTCG | BglII |
| P2F1 | CGCGGATCCTATAACAACGAAGGGACTAACG | BamHI |
| P2R1 | CGCGTCGACCAAATGATTAGAAGTAAACGC | SalI |
| P2∆1-4I_F | CGCGGATCCAACTACAAATATAACGAAGCTGACG | BamHI |
| P2∆1-4I_R | GTCACTATTGTTGAGAACGCC | Not present |
| P2∆5-8I_F | GTGTCTCTAGATAGTGGCTATGC | Not present |
| P2∆5-8I_R | CGCGTCGACGTAGTTAGTTCTACCATAAGCAATTTTTGC | SalI |
| P2∆L1I_F | CGCAGATCTGGTTCACGTTTCCACATTAAAGC | BglII |
| P2∆L1I_R | CGCAGATCTACTTTGTTCTGCGATAATGCTTAAAC | BglII |
| P2∆L2I_F | CGCAGATCTGGTGATATTACAAGCAAATATGCTTATG | BglII |
| P2∆L2I_R | CGCAGATCTTTTTGTAACAAAACGAGTTTCTAAATAA | BglII |
| P2∆L3I_F | CGCAGATCTGGTAATACCGTTGGCTATACTTTT | BglII |
| P2∆L3I_R | CGCAGATCTCGCACGACCAAGTTTTACTTCAC | BglII |
| P2∆L4I_F | CGCAGATCTATAGGTGAAATCAATAATGGAATTCAAG | BglII |
| P2∆L4I_R | CGCAGATCTCTTTTGTGCTAATAAATAATTAGCGCC | BglII |
| L4-F1 | GCGAGATCTGCTGTTGTCGGCAAGCCCGGATCG | BglII |
| L4-R1 | GCGGGATCCAGCCGGCGTATAGGCGGACTTGCTG | BamHI |
| L4-F2 | GCGAGATCTGCTTTGTTTTTGACGGCTCGC | BglII |
| L4-R2 | GCGAGATCTATCCGCTTCACCGCCCCG | BglII |
| F1 | CACGCTTTATAGTGATAGCC | Not present |
| mP2_R | GACCAAACTAGCGTTGAAACC | Not present |
| OmgF | GCGGATCCAAGTGCGGTCAAGCAAGGCGAATCGAAAGAT | BamHI |
| OmgR | GCGGATCCTCTAACAAATGATTAGAAGTAAACGC | BamHI |
| MegaR | GCAAGTGTTTTTTTCATAATTTGTATTCC | Not present |
| ∆P2F | CGCAGATCTGCGTTTACTTCTAATCATTTG | BglII |
| P2FR | CGCGCGGCCGCGCGGTAAAAATTATGCGTGAA | NotI |

*^a^* All primers were designed from the *N. meningitidis* MC58 or *H. influenzae* Rd KW20 genome sequences. Underlined sequences identify restriction enzyme sites.
